# Supplementary material for: Contamination detection and microbiome exploration with GRIMER
Source: Gigascience. 2023 Mar 30;12:giad017. doi: 10.1093/gigascience/giad017 (PMC10061425; doi:10.1093/gigascience/giad017)
Supplement: giad017_Response_to_Reviewer_Comments_Original_Submission [file giad017_response_to_reviewer_comments_original_submission.pdf]

Author's Response To Reviewer Comments

Close

Point-by-point responses to the reviewers are addressed below. In the main manuscript, updated text is marked in red.

Reviewer #1: Piro and Renard present GRIMER, which is a bioinformatics tool for summarizing microbiome taxonomic data in various ways, with the main purpose of identifying putatively contaminant taxa. The authors convincingly argue that there is great value in looking at several different aspects of a dataset when determining which taxa are potential contaminants. I think this tool could potentially be very useful for the field, but I think at the moment there are several places where users might be confused and perhaps be overwhelmed without more documentation.

The main point of confusion I'm concerned about is regarding the "common contaminants". It's not convincing that you can just classify a taxon as a contaminant regardless of what environment is being profiled. Also, under this approach, if a taxon is identified once as a contaminant in an earlier study, would it then be classified as a contaminant in all datasets processed by GRIMER? This would mean that a lot of high-abundance taxa in certain environments would be wrongly thrown out. For instance, you can imagine high-abundance taxa on the human skin might be more likely to be contaminants during sequencing preparation, but of course many researchers are very interested in profiling the skin microbiome. I think the authors realize this, but I'm concerned that typical users may not appreciate this point. I think explicit discussion of this point in the discussion is needed and also an example of how this might look in practice (e.g., if skin microbiome samples were input to
GRIMER, as part of a larger tutorial that could be online [see next point], would help avoid this mistake).

--- Answer ---
Thank you for the valuable suggestion. In our own experience, contamination can be very difficult to detect and looking at many aspects can lead to better decision. The use of the common contaminants should be just one evidence supporting contamination. We clarified the intended use of the common contaminant list in the main article:
“The idea behind compiling this list is to detect which taxa is the most recurrently identified as contaminant in diverse conditions, providing a guideline and consensus for further studies. Entries on this list are not strictly considered a contaminant and should not be used alone to define contamination in a study. However, it serves as an additional evidence supporting it, especially if entries are highly recurrent (Table 4) and corroborate with additional lines of evidence.”

We also expanded and clarified the use of such annotations in the discussions:
“In addition to the GRIMER software, we compiled and provided in this work a list of common taxa contaminants based on 22 publications (Table 3). Many of the reported contaminants are recurrent in diverse studies, pointing to a consensus for some taxa (Table 4) as a probable contaminant. Taxa in this list cannot be strictly considered a contaminant by itself. However, they can corroborate suspicious contamination discovered via several other lines of evidence without the extra effort of researching the literature. The presented list is not comprehensive but a first step to centralize and standardize re-occurring contaminants described in the literature. We expect this list to incrementally grow overtime as more evidence of kit and laboratory contamination becomes available. The information of common contaminants is a valuable resource to aid contamination detecting and we are willing to keep and extend it. Improvements to the list and suggestions of further candidate taxa can be provided via the GRIMER repository at https://github.com/pirovc/grimer/. As a future work, the list can be associated with study details as biome, extraction kit and methodology to be further queried and integrated in more details.

Additionally to the aforementioned common contaminants, GRIMER can also use general lists of custom organisms to annotate samples. In this work and by default, human-related organisms commonly occurring

in human skin, oral and nasal cavities as well as face and other human limbs are used since they can be external sources of contamination. Those lists can be easily provided as taxonomic identifiers or names to GRIMER. If the target study conflicts with any of those environments (e.g. study of human skin), one could simply remove the related entries from the configuration files. More details and examples on how to perform this can be found in the online documentation."

We also included in the newly created documentation instructions on how to deal with overlapping studies based on our contamination lists:
"Common contaminants compiled from the literature and human-related possible sources of contamination are available in the GRIMER repository. For more information, please refer to the pre-print. If the target study overlaps with some of those annotation (e.g. study of human skin), related entries can be easily removed from the provided files to not generate redundant annotations."

The authors do a great job of walking through some results in the text, but more documentation is needed for the reports. The authors should include a basic tutorial that provides example input files and then walks through each individual tab. This could done all through text with screenshots of the GRIMER, or perhaps with a video tutorial. In addition, for someone just opening the example reports, I'm sure they will be wondering what data was produced by GRIMER (e.g., they might wrongly think GRIMER did the taxonomic classiciation) and what data was needed as input.

--- Answer ---
An online documentation page was created for running GRIMER as well as an user manual for GRIMER reports, with a walk through each tab and plot. They can be found in the following page:
https://pirovc.github.io/grimer/

The authors should expand on how the correlation step is used to identify contaminants. There is great interest in identifying clusters of co-occurring taxa, so identifying a cluster of 9 genera in Figure 5 doesn't seem like evidence of contamination to me. Perhaps it is when considered with other lines of evidence though, but this should be made clearer. Currently this legend implies that it alone points to reagent-derived contamination

--- Answer ---
Indeed, the correlation alone can not define contamination and the information was misleading. This is now fixed and clarified in the main text:

"Symmetric proportionality coefficient (rho correlation) between top 50 most abundant genera in the KatharoSeq data. Positive correlation values (between 0 and 1) are displayed in red. Negative correlation values (between -1 and 0) are displayed in blue. Highly correlated matrix among 9 genera (dark red) points to reagent-derived contamination, when considered with other lines of evidence (Figure 6)"

The figure text needs to be increased in size. Using more panels split across additional rows and removing unnecessary info (e.g., not all control categories need to be shown in Figure 1) would make these figures easier to interpret. I realize that you were hoping to use the raw GRIMER figures, but based on the current display items it does not seem like they are publication ready.

--- Answer ---
All figures were re-generated, with increased text size. Some of them were split in further panels for better visualization.

The acronym WGS generally refers to "whole genome sequencing" (i.e., for single isolate organisms) not "whole metagenome sequencing". The standard acronym for the latter case would be "MGS", for "metagenomics". Also, the term "shotgun metagenomics sequencing" is mostly commonly used in this context, I've never come across "whole metagenome sequencing" before. Either way, "WGS" will mislead

casual readers with the current usage, so this should be changed on your website and in the manuscript.

--- Answer ---
The term was changed in the manuscript to MGS, referring to metagenomics as well as in the pictures and reports.

The taxa parsing capabilities sound like they will save a lot of tedious, manual data mapping! Just checking - how does it perform with new taxa names / typos?

--- Answer ---
The taxonomy parsing and conversion are enabled with MultiTax (https://github.com/pirovc/multitax). New names are not a problem, since GRIMER will always download the latest taxonomy available when running and automatically find and update them. Typos are not covered and entries will still be included in the report, just without any taxonomic connections (annotations, MGnify).

Text edits

L11 - "are challenging task" should be "is challenging"
Done

L12 - can remove "by design"
Done

L12 - "helping to" should be "to help"
Done

L13 - "can potentially be a source" I think should be "that could reflect"
Changed to: "that could potentially be contamination"

L14 - "evidences" should be "evidence"
Done

L13 + L14 - Unclear what is meant by "external evidences, aggregation of methods and data and common contaminant" - should be clarified
Changed to "Additionally, external evidence, like aggregation of several contamination detection methods and the use of common contaminants reported in the literature could help to discover and mitigate contamination."

L15 - "that perform" should be "that performs"
Done

L17 - "towards contamination detection" should be something like "to help detect contamination"
Done

L41 - "hypothesis" should be "hypotheses"
Done

L42/43 - "analysis can hardly be fully" should be something like "the required analysis is difficult to fully…"
Changed to: "After measurements are obtained, hypotheses are validated through data mining and statistical analysis. This step is mostly exploratory and specific to the hypotheses and research questions pursued and the required analysis is difficult to be fully automatized."

L56 - "technicians body" should be "a technician's body"
Done

L60 - "strongly affects environmental" should be "especially environmental," (note comma)
Done

L64 - "ideal scenario for an" should be "an ideal scenario for"
Done

L67 - "not to bias measurements and not to" should be reworded, possibly as: "to not bias measurements and to ensure that bias is not propagated into databases"
Done

L75 - "were proposed. They are " should be "have been proposed. These are"
Done

L77 - "among others" should be ", and others" (note comma)
Done

L79 - "increase in costs" should be "the required increase in costs"
Done

L88 - add "a" before focus
Done

L90, L196, L265, and elsewhere - "evidences" should be "evidence"
Done

L99, L104, L117, and possibly elsewhere - "analysis" should be "analyses" (when plural)
Done

L106 - "each samples/compositions" should be "each sample/composition"
Done

L110 - add "a" before taxonomy database and "the" before "DNA concentration"
Done

L132 - "specially" should be "especially"
Done

L134 - remove "a" before "the"
Done

L151 - add "of" after "thousands"
Done

L182 - "is" should be "are"
Done

L196 - "evidences" should be "evidence". And rather than "Evidences towards" it would be correct to say "Evidence for" or "Evidence supporting"
Done

L208 - add "the" before "overall"
Done

L246/247 - "generated several studies and investigations" should be something like "motivated several investigations"
Changed to: "The attempt to detect and describe a possible human placental microbiome has motivated several studies and investigations"

L248 - should be something like "from the maternal and fetal sides"
Done

L279 - remove "a"
Done (L278)

L280 - Add "the" before "Jet"
Done

L284 - capitalize "Qiita" and re-word "Pick closed-reference OTUs with 97% annotated with greengenes taxonomy"
Done and changed to: “We downloaded the OTU table and metadata from KatharoSeq evaluations for the 16S rRNA analyses available in Qiita in the following configuration: reads trimmed at 150bp and classified using closed-reference OTUs clustered at 97% similarity annotated with the greengenes taxonomy.”

L293 - Should be "Furthermore" rather than "Further"
Done

L295 - I think it should be "with low and high human exposure, respectively"? Or do you mean they both have highly variable exposure?
Done

L297 - "could be a also an" should be "could be driven by an"
Done

L300 - "against" should be "and"
Done

L304 - "correlated genus" should be "correlated genera" (and in other cases, such as in the Fig 5 and 6 legends, where "genus" should be plural version, i.e., "genera")
Done in all occurrences

L305 - "Such pattern" should be "Such a pattern"
Done

L307 - Should be "groups" rather than "organisms groups", or just "genera" as I believe each is a genus
Done

L313 - Remove "a"
Done

Fig 5 legend: "point" should be "points"
Done

Fig 6 legend: "taxa is abundant" should be "This taxon is abundant" and "inversely correlate" should be "inversely correlated". "a contamination evidence" should be "potential contamination"
Done

Reviewer #2: Piro and Renard introduce GRIMER, a tool that automates microbiome-related analyses and creates rich, offline-supported report that can be shared with collaborators or hosted online. I think that they gave a great summary of the problem of contamination in the microbiome field, and clearly explain the gap that their software fills. They exhibit GRIMER on previously published datasets, which are available to view online. Overall, I'm very impressed with the dashboard—it looks great, is easy to explore datasets, and highly portable. I can certainly see myself using GRIMER on some of my future datasets, and I have no

doubt that it can be a valuable tool for others in the field. I do however think that the documentation and usability of the tool can be improved, and I give some suggestions below. Addressing these issues will, in my opinion, lead to a wider adoption of the tool by researchers in the field.

Usability:
I managed to test GRIMER on a 16S amplicon dataset, but given the sparsity of the documentation, this took me a little longer than expected (in addition to quite a few steps), and I think that there are improvements that could be made to make it easier for people to use GRIMER from formats that people commonly generate.

--- Answer ---
An online documentation page was created for running GRIMER as well as an user manual for GRIMER reports, with a walk through each tab and plot. They can be found in the following page:
https://pirovc.github.io/grimer/

For example, QIIME2 is perhaps the most used 16S amplicon analysis pipeline, so the ability to import directly from .qza files (e.g. table.qza, taxonomy.qza) would give GRIMER much greater reach. If this is beyond the scope to incorporate within the GRIMER codebase, at least provide the exact code needed in the documentation for people to export their .qza files to files compatible with GRIMER.
Likewise from phyloseq, a commonly used R package for microbiome analyses. Could some documentation/code be added about how best to export phyloseq objects to a format that GRIMER can handle?

--- Answer ---
Thanks for the suggestion. We included in the manual a guide on how to run GRIMER from commonly used tools, with QIIME2 and phyloseq included.

I mostly analyse shotgun metagenomic datasets (genome-resolved), and I foresee more users using these types of data in the future. Therefore, the ability to parse gtdb-tk outputs directly would be very helpful. Perhaps have a flag --gtdb that parses the 'gtdbtk.bac120.summary.tsv' and 'gtdbtk.ar53.summary.tsv' files.
Following on from this, CoverM (https://github.com/wwood/CoverM) is quite commonly used for generating final MAG count tables (.tsv), so the ability to import them directly would be a really nice quality-of-life addition, and something that would not require much coding to accomplish.
I believe that these adjustments will make the tool far more accessible for everyday users and increase the adoption of GRIMER by the wider community.

--- Answer ---
Thanks for the suggestion. We included in the documentation several ways to run GRIMER via .biom or .tsv files. Further, we made all base files from the analysis of the manuscript available and provided the code used to generate them (https://pirovc.github.io/grimer/examples/). We believe that those methods are generalized enough and will cover the usage for users comming from gtdb-tk and CoverM.

For the actual report, if possible, I would like the ability to export ASVs/features/MAGs from the report that the user thinks are contaminants. This could be a list that the user could copy/paste, or the direct export of a .txt/.tsv. Perhaps the user could tick a box next to the ASVs/features/MAGs to save them to a list/viewer? The reason for this is that the logical next step I see after using GRIMER is to go back to your dataset and filter out the putative contaminant ASVs/features/MAGs. Being able to produce such a list will make subsequent filtering by the user easier.

--- Answer ---
Thanks for the valuable request. An export button was added to the Overview and Samples panels, where the user can easily export selected or all items of the tables for further usage.

I couldn't get decontam to work with my dataset, here was the error:
raise KeyError(f"None of [{key}] are in the [{axis_name}]")
KeyError: "None of [Float64Index([nan, nan, nan, nan, nan, nan, nan, nan, nan, nan, nan, nan, nan,\n nan, nan, nan, nan, nan, nan, nan, nan, nan, nan, nan, nan, nan,\n nan, nan, nan, nan],\n dtype='float64')] are in the [index]"

I can post this as an issue on the repo if you'd like.

--- Answer ---
It would be nice to have it reported in the repository so I can detect and fix the possible error. Instead of re-implementing it, GRIMER runs DECONTAM directly in R to keep compatibility and avoid any implementation differences. This is important due to the different way numeric values are treated in R and python. However, this brigde between languages can be tricky and may bring some issues. A bug report would be very helpful to improve the conversion code for general use cases.

Regarding the specification of negative and positive controls in the config.yaml, would it be possible for this to be implemented from the executable? For example, there could be a flag '--control-column' that specifies the column in the user's metadata file. '--control-column control' would parse the 'control' metadata column, and for cases where are values 'negative', 'positive' assign them automatically. This is just a suggestion that could make it a bit easier for users to set control samples, rather than having to create a new .txt file and change the config.yml.

--- Answer ---
Thanks for the suggestion. It is now possible to provide which samples are controls defining a metadata field and value(s) in the config.yml file in addition to a file containing the sample identifiers. Detailed information on how to set-up the controls are now in the manual: https://pirovc.github.io/grimer/config/

Dependencies:
When installing via conda, I ran into the following error:
ImportError: cannot import name 'PearsonRConstantInputWarning' from 'scipy.stats'

It seems that this can't be imported from later versions of scipy, but I managed to fix it by forcing scipy=1.8.1. You should be able to force this version in the conda recipe.

--- Answer ---
I tried different versions of scipy (1.9.3 and 1.10.0) and could not replicate this error using all GRIMER features. It would be nice to have it reported in the repository with your data so I can detect and fix the possible error.

Minor grammar:

Line 16: replace 'perform' with 'performs'
Done

Line 50: 'found in the [9]'
Changed to: “A comparison among many of those methods and their functionalities can be found in a recent review [9].”

Line 56: replace 'as technicians body' with 'microbes from laboratory technicians'
Done

Line 60: I would remove the 'environmental' adjective here, as contamination affects all low-biomass samples.

Done

Line 63: I would use 'samples' in place of 'environments' here. You may also consider suggesting that some samples may even contain no microbial DNA. E.g. replace 'low amounts of' with 'little to no'.
Done

Line 64: Replace 'ideal scenario for an exogenous contaminants' with 'an ideal scenario for exogenous contaminants'.
Done

Line 72: perhaps consider referencing decontam here.
Done

Line 79: replace 'due to increase in costs' with 'due to the increase in cost associated with their inclusion'.
Changed to: “Further, many studies do not include or have limited number of control samples due to the required increase in costs.”

Line 81: Consider referencing first author's last name, e.g. 'Moreover, XXX et al. [45] reported…'
Done

Line 88: remove 'outcomes'
Done

Close
