# Supplementary material for: Contamination detection and microbiome exploration with GRIMER
Source: Gigascience. 2023 Mar 30;12:giad017. doi: 10.1093/gigascience/giad017 (PMC10061425; doi:10.1093/gigascience/giad017)
Supplement: giad017_Response_to_Reviewer_Comments_Revision_1 [file giad017_response_to_reviewer_comments_revision_1.pdf]

Author's Response To Reviewer Comments

Close

Dear Hans Zauner,

I addressed all points requested in the main text file of the manuscript. Please let me know if there is anything else necessary or if something is not adequate.

Best Regards,
Vitor C. Piro

Close
