# Supplementary material for: Contamination detection and microbiome exploration with GRIMER
Source: Gigascience. 2023 Mar 30;12:giad017. doi: 10.1093/gigascience/giad017 (PMC10061425; doi:10.1093/gigascience/giad017)
Supplement: giad017_Response_to_Reviewer_Comments_Revision_2 [file giad017_response_to_reviewer_comments_revision_2.pdf]

Author's Response To Reviewer Comments

Close

Dear Hans Zauner,

I adapted the citations as requested and move the identifiers to the tabular section. I made sure the .bib file has the correct information once the manuscript is used in the proper latex layout. Please let me know if something else is not proper.

Best Regards,
Vitor C. Piro

Close
