# Supplementary material for: Contamination detection and microbiome exploration with GRIMER
Source: Gigascience. 2023 Mar 30;12:giad017. doi: 10.1093/gigascience/giad017 (PMC10061425; doi:10.1093/gigascience/giad017)
Supplement: giad017_Reviewer_1_Report_Original_Submission — Gavin M Douglas -- 11/26/2022 Reviewed [file giad017_reviewer_1_report_original_submission.pdf]

Reviewer Report

Title: Contamination detection and microbiome exploration with GRIMER

Version: Original Submission **Date:** 11/26/2022

Reviewer name: Gavin M Douglas

Reviewer Comments to Author:

Piro and Renard present GRIMER, which is a bioinformatics tool for summarizing microbiome taxonomic data in various ways, with the main purpose of identifying putatively contaminant taxa. The authors convincingly argue that there is great value in looking at several different aspects of a dataset when determining which taxa are potential contaminants. I think this tool could potentially be very useful for the field, but I think at the moment there are several places where users might be confused and perhaps be overwhelmed without more documentation.The main point of confusion I'm concerned about is regarding the "common contaminants". It's not convincing that you can just classify a taxon as a contaminant regardless of what environment is being profiled. Also, under this approach, if a taxon is identified once as a contaminant in an earlier study, would it then be classified as a contaminant in all datasets processed by GRIMER? This would mean that a lot of high-abundance taxa in certain environments would be wrongly thrown out. For instance, you can imagine high-abundance taxa on the human skin might be more likely to be contaminants during sequencing preparation, but of course many researchers are very interested in profiling the skin microbiome. I think the authors realize this, but I'm concerned that typical users may not appreciate this point. I think explicit discussion of this point in the discussion is needed and also an example of how this might look in practice (e.g., if skin microbiome samples were input to GRIMER, as part of a larger tutorial that could be online [see next point], would help avoid this mistake).The authors do a great job of walking through some results in the text, but more documentation is needed for the reports. The authors should include a basic tutorial that provides example input files and then walks through each individual tab. This could done all through text with screenshots of the GRIMER, or perhaps with a video tutorial. In addition, for someone just opening the example reports, I'm sure they will be wondering what data was produced by GRIMER (e.g., they might wrongly think GRIMER did the taxonomic classiciation) and what data was needed as input.The authors should expand on how the correlation step is used to identify contaminants. There is great interest in identifying clusters of co-occurring taxa, so identifying a cluster of 9 genera in Figure 5 doesn't seem like evidence of contamination to me. Perhaps it is when considered with other lines of evidence though, but this should be made clearer. Currently this legend implies that it alone points to reagent-derived contaminationThe figure text needs to be increased in size. Using more panels split across additional rows and removing unnecessary info (e.g., not all control categories need to be shown in Figure 1) would make these figures easier to interpret. I realize that you were hoping to use the raw GRIMER figures, but based on the current display items it does not seem like they are publication ready.The acronym WGS generally refers to "whole genome sequencing" (i.e., for single isolate organisms) not "whole metagenome sequencing". The standard acronym for the latter case would be "MGS", for "metagenomics". Also, the term "shotgun metagenomics sequencing" is mostly commonly used in this

context, I've never come across "whole metagenome sequencing" before. Either way, "WGS" will mislead casual readers with the current usage, so this should be changed on your website and in the manuscript.The taxa parsing capabilities sound like they will save a lot of tedious, manual data mapping! Just checking - how does it perform with new taxa names / typos?Text editsL11 - "are challenging task" should be "is challenging"L12 - can remove "by design"L12 - "helping to" should be "to help"L13 - "can potentially be a source" I think should be "that could reflect"L14 - "evidences" should be "evidence"L13 + L14 - Unclear what is meant by "external evidences, aggregation of methods and data and common contaminant" - should be clarifiedL15 - "that perform" should be "that performs"L17 - "towards contamination detection" should be something like "to help detect contamination"L41 - "hypothesis" should be "hypotheses"L42/43 - "analysis can hardly be fully" should be something like "the required analysis is difficult to fully…"L56 - "technicians body" should be "a technician's body"L60 - "strongly affects environmental" should be "especially environmental," (note comma)L64 - "ideal scenario for an" should be "an ideal scenario for"L67 - "not to bias measurements and not to" should be reworded, possibly as: "to not bias measurements and to ensure that bias is not propagated into databases"L75 - "were proposed. They are " should be "have been proposed. These are"L77 - "among others" should be ", and others" (note comma)L79 - "increase in costs" should be "the required increase in costs"L88 - add "a" before focusL90, L196, L265, and elsewhere - "evidences" should be "evidence"L99, L104, L117, and possibly elsewhere - "analysis" should be "analyses" (when plural)L106 - "each samples/compositions" should be "each sample/composition"L110 - add "a" before taxonomy database and "the" before "DNA concentration"L132 - "specially" should be "especially"L134 - remove "a" before "the"L151 - add "of" after "thousands"L182 - "is" should be "are"L196 - "evidences" should be "evidence". And rather than "Evidences towards" it would be correct to say "Evidence for" or "Evidence supporting"L208 - add "the" before "overall"L246/247 - "generated several studies and investigations" should be something like "motivated several investigations"L248 - should be something like "from the maternal and fetal sides"L279 - remove "a"L280 - Add "the" before "Jet"L284 - capitalize "Qiita" and re-word "Pick closed-reference OTUs with 97% annotated with greengenes taxonomy"L293 - Should be "Furthermore" rather than "Further"L295 - I think it should be "with low and high human exposure, respectively"? Or do you mean they both have highly variable exposure?L297 - "could be a also an" should be "could be driven by an"L300 - "against" should be "and"L304 - "correlated genus" should be "correlated genera" (and in other cases, such as in the Fig 5 and 6 legends, where "genus" should be plural version, i.e., "genera")L305 - "Such pattern" should be "Such a pattern"L307 - Should be "groups" rather than "organisms groups", or just "genera" as I believe each is a genusL313 - Remove "a"Fig 5 legend: "point" should be "points"Fig 6 legend: "taxa is abundant" should be "This taxon is abundant" and "inversely correlate" should be "inversely correlated". "a contamination evidence" should be "potential contamination"

Level of Interest

Please indicate how interesting you found the manuscript: Choose an item.

Quality of Written English

Please indicate the quality of language in the manuscript: Choose an item.

Declaration of Competing Interests

Please complete a declaration of competing interests, considering the following questions:

- Have you in the past five years received reimbursements, fees, funding, or salary from an organisation that may in any way gain or lose financially from the publication of this manuscript, either now or in the future?
- Do you hold any stocks or shares in an organisation that may in any way gain or lose financially from the publication of this manuscript, either now or in the future?
- Do you hold or are you currently applying for any patents relating to the content of the manuscript?
- Have you received reimbursements, fees, funding, or salary from an organization that holds or has applied for patents relating to the content of the manuscript?
- Do you have any other financial competing interests?
- Do you have any non-financial competing interests in relation to this paper?

If you can answer no to all of the above, write 'I declare that I have no competing interests' below. If your reply is yes to any, please give details below.

I declare that I have no competing interests

I agree to the open peer review policy of the journal. I understand that my name will be included on my report to the authors and, if the manuscript is accepted for publication, my named report including any attachments I upload will be posted on the website along with the authors' responses. I agree for my report to be made available under an Open Access Creative Commons CC-BY license (http://creativecommons.org/licenses/by/4.0/). I understand that any comments which I do not wish to be included in my named report can be included as confidential comments to the editors, which will not be published.

Choose an item.

To further support our reviewers, we have joined with Publons, where you can gain additional credit to further highlight your hard work (see: https://publons.com/journal/530/gigascience). On publication of this paper, your review will be automatically added to Publons, you can then choose whether or not to claim your Publons credit. I understand this statement.

Yes Choose an item.
