# Supplementary material for: Contamination detection and microbiome exploration with GRIMER
Source: Gigascience. 2023 Mar 30;12:giad017. doi: 10.1093/gigascience/giad017 (PMC10061425; doi:10.1093/gigascience/giad017)
Supplement: giad017_Reviewer_2_Report_Original_Submission — Raphael Eisenhofer -- 11/28/2022 Reviewed [file giad017_reviewer_2_report_original_submission.pdf]

Reviewer Report

Title: Contamination detection and microbiome exploration with GRIMER

Version: Original Submission **Date:** 11/28/2022

Reviewer name: Raphael Eisenhofer

Reviewer Comments to Author:

Piro and Renard introduce GRIMER, a tool that automates microbiome-related analyses and creates rich, offline-supported report that can be shared with collaborators or hosted online. I think that they gave a great summary of the problem of contamination in the microbiome field, and clearly explain the gap that their software fills. They exhibit GRIMER on previously published datasets, which are available to view online. Overall, I'm very impressed with the dashboardâ€"it looks great, is easy to explore datasets, and highly portable. I can certainly see myself using GRIMER on some of my future datasets, and I have no doubt that it can be a valuable tool for others in the field. I do however think that the documentation and usability of the tool can be improved, and I give some suggestions below. Addressing these issues will, in my opinion, lead to a wider adoption of the tool by researchers in the field.Usability:I managed to test GRIMER on a 16S amplicon dataset, but given the sparsity of the documentation, this took me a little longer than expected (in addition to quite a few steps), and I think that there are improvements that could be made to make it easier for people to use GRIMER from formats that people commonly generate.For example, QIIME2 is perhaps the most used 16S amplicon analysis pipeline, so the ability to import directly from .qza files (e.g. table.qza, taxonomy.qza) would give GRIMER much greater reach. If this is beyond the scope to incorporate within the GRIMER codebase, at least provide the exact code needed in the documentation for people to export their .qza files to files compatible with GRIMER.Likewise from phyloseq, a commonly used R package for microbiome analyses. Could some documentation/code be added about how best to export phyloseq objects to a format that GRIMER can handle?I mostly analyse shotgun metagenomic datasets (genome-resolved), and I foresee more users using these types of data in the future. Therefore, the ability to parse gtdb-tk outputs directly would be very helpful. Perhaps have a flag --gtdb that parses the 'gtdbtk.bac120.summary.tsv' and 'gtdbtk.ar53.summary.tsv' files.Following on from this, CoverM (https://github.com/wwood/CoverM) is quite commonly used for generating final MAG count tables (.tsv), so the ability to import them directly would be a really nice quality-of-life addition, and something that would not require much coding to accomplish.I believe that these adjustments will make the tool far more accessible for everyday users and increase the adoption of GRIMER by the wider community.For the actual report, if possible, I would like the ability to export ASVs/features/MAGs from the report that the user thinks are contaminants. This could be a list that the user could copy/paste, or the direct export of a .txt/.tsv. Perhaps the user could tick a box next to the ASVs/features/MAGs to save them to a list/viewer? The reason for this is that the logical next step I see after using GRIMER is to go back to your dataset and filter out the putative contaminant ASVs/features/MAGs. Being able to produce such a list will make subsequent filtering by the user easier.I couldn't get decontam to work with my dataset, here was the error:raise KeyError(f"None of [{key}] are in the [{axis_name}]")KeyError: "None of [Float64Index([nan, nan, nan,

nan, nan, nan, nan, nan, nan, nan, nan, nan, nan,\n nan, nan, nan, nan, nan, nan, nan, nan, nan, nan, nan, nan, nan,\n nan, nan, nan, nan],\n dtype='float64')] are in the [index]"I can post this as an issue on the repo if you'd like.Regarding the specification of negative and positive controls in the config.yaml, would it be possible for this to be implemented from the executable? For example, there could be a flag '--control-column' that specifies the column in the user's metadata file. '--control-column control' would parse the 'control' metadata column, and for cases where are values 'negative', 'positive' assign them automatically. This is just a suggestion that could make it a bit easier for users to set control samples, rather than having to create a new .txt file and change the config.yml.Dependencies:When installing via conda, I ran into the following error:ImportError: cannot import name 'PearsonRConstantInputWarning' from 'scipy.stats'It seems that this can't be imported from later versions of scipy, but I managed to fix it by forcing scipy=1.8.1. You should be able to force this version in the conda recipe.Minor grammar:Line 16: replace 'perform' with 'performs'Line 50: 'found in the [9]'Line 56: replace 'as technicians body' with 'microbes from laboratory technicians'Line 60: I would remove the 'environmental' adjective here, as contamination affects all low-biomass samples.Line 63: I would use 'samples' in place of 'environments' here. You may also consider suggesting that some samples may even contain no microbial DNA. E.g. replace 'low amounts of' with 'little to no'.Line 64: Replace 'ideal scenario for an exogenous contaminants' with 'an ideal scenario for exogenous contaminants'.Line 72: perhaps consider referencing decontam here.Line 79: replace 'due to increase in costs' with 'due to the increase in cost associated with their inclusion'.Line 81: Consider referencing first author's last name, e.g. 'Moreover, XXX et al. [45] reported…'Line 88: remove 'outcomes'

Level of Interest

Please indicate how interesting you found the manuscript: Choose an item.

Quality of Written English

Please indicate the quality of language in the manuscript: Choose an item.

Declaration of Competing Interests

Please complete a declaration of competing interests, considering the following questions:

- Have you in the past five years received reimbursements, fees, funding, or salary from an organisation that may in any way gain or lose financially from the publication of this manuscript, either now or in the future?
- Do you hold any stocks or shares in an organisation that may in any way gain or lose financially from the publication of this manuscript, either now or in the future?
- Do you hold or are you currently applying for any patents relating to the content of the manuscript?
- Have you received reimbursements, fees, funding, or salary from an organization that holds or has applied for patents relating to the content of the manuscript?

- Do you have any other financial competing interests?
- Do you have any non-financial competing interests in relation to this paper?

If you can answer no to all of the above, write 'I declare that I have no competing interests' below. If your reply is yes to any, please give details below.

I declare that I have no competing interests.

I agree to the open peer review policy of the journal. I understand that my name will be included on my report to the authors and, if the manuscript is accepted for publication, my named report including any attachments I upload will be posted on the website along with the authors' responses. I agree for my report to be made available under an Open Access Creative Commons CC-BY license (http://creativecommons.org/licenses/by/4.0/). I understand that any comments which I do not wish to be included in my named report can be included as confidential comments to the editors, which will not be published.

Choose an item.

To further support our reviewers, we have joined with Publons, where you can gain additional credit to further highlight your hard work (see: https://publons.com/journal/530/gigascience). On publication of this paper, your review will be automatically added to Publons, you can then choose whether or not to claim your Publons credit. I understand this statement.

Yes Choose an item.
